# Template Directed Reversible Photochemical Ligation of Oligodeoxynucleotides

**DOI:** 10.3390/molecules17010163

**Published:** 2011-12-27

**Authors:** Shigetaka Nakamura, Shinzi Ogasawara, Shigeo Matuda, Isao Saito, Kenzo Fujimoto

**Affiliations:** 1 School of Materials Science, Japan Advanced Institute of Science and Technology, Nomi, Ishikawa 923-1292, Japan; 2 Department of Synthetic Chemistry and Biological Chemistry, Factly of Enginnering, Kyoto University, Kyoto 606-8501, Japan; 3 Research Center for Bio-Architecture, Japan Advanced Institute of Science and Technology, Nomi, Ishikawa 923-1292, Japan

**Keywords:** photochemical ligation, 5-carboxyvinyldeoxyuridine, 5-vinyldeoxyuridine, DNA nanotechnology

## Abstract

We demonstrated that 5-vinyldeoxyuridine (^V^U) and 5-carboxyvinyldeoxyuridine (^CV^U) can be used to photoligate a longer oligonucleotide (ODN) from smaller ODNs on a template. By performing irradiation at 366 nm, these artificial nucleotides make photoligated ODNs with high efficiency without any side reactions. Moreover, by performing irradiation at 312 nm, these photoligated ODNs were reversed to the original ODN. ^V^U needs to be irradiated 366 nm for 6 h, but ^CV^U needs to be irradiated at 366 nm for 15 min. Finally, we made a self-assembled structure with an ODN containing ^CV^U and observed the photoligated ODN by photoirradiation.

## 1. Introduction

**Scheme 1:**
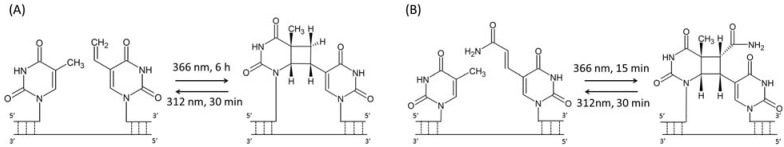
Scheme of photochemical reaction (**A**) ^V^U; (**B**) ^CV^U.

There are many methods of template-directed chemical ligation of oligonucleotides (ODNs) via a native phosphodiester bond [1] or non-native linking [2]. Template-directed chemical ligation can ligate not only DNA, but also other biomolecules such as protein-like molecules [3]. Template directed synthesis has been used for DNA nanotechnology [4], the selection of amplifiable small-molecule libraries [5], the release of drugs [6], and as a diagnostic means of detecting the presence of the nucleic acid template [7]. One method of template-directed chemical ligation is non-enzymatic chemical ligation [8,9]. This method was researched for new gene manipulation and as a design method for nanostructures, because it does not have restrictions of a substrate and reaction conditions are suitable for enzymatic reactions. In particular, photochemical ligation has many useful characteristics. For example, there is no need to add other reagents and the ligation reaction is easily regulated by irradiation wavelength and intensity. However, there are only a few methods of performing photochemical reactions [10,11,12]. Previously reported methods for DNA photochemical ligation are thymine dimer formation [10], photoreactions of DNA containing appended stilbenes [11] and ligated DNA using anthracene [12]. However, these methods have a serious problem for practical utilization, such as the low yields of photochemical ligation products and the use of short wavelengths that injure other biological components. We reported that 5-vinyldeoxyuridine (VU) can be used to photolink a longer ODN from five smaller identical ODNs on a template with high efficiency without any side reactions by photoirradiation at 366 nm [13]. This reaction ligated T and VU via [2+2] photocyclization [14]. Moreover, we have reported 5-carboxyvinyldeoxyuridine (CVU) [15] an artificial nucleotide which is photoresponsive. The photochemical reaction of VU and CVU is shown in Scheme 1. In this study, we synthesized a longer ODN from four smaller different ODNs by photochemical ligation with VU and 5-vinlycytosine (VC) [16]. Next, we conducted the same experiment using CVU and confirmed the template dependence. Finally, we researched photochemical ligation activity using CVU.

## 2. Results and Discussion

**Figure 1:**
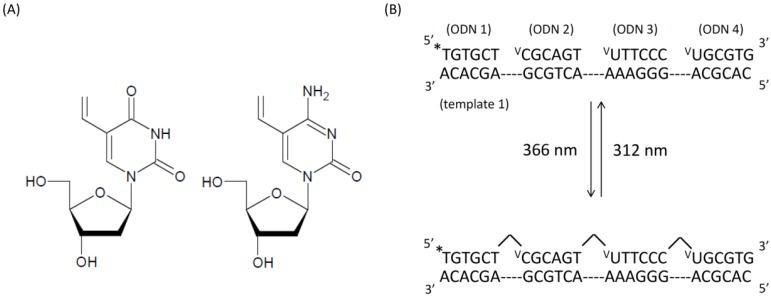
(**A**) Chemical structure of ^V^U and ^V^C nucleoside; (**B**) Schematic illustration for reversible photoligation with ^V^U and ^V^C. *: ^32^P label.

**Scheme 2:**
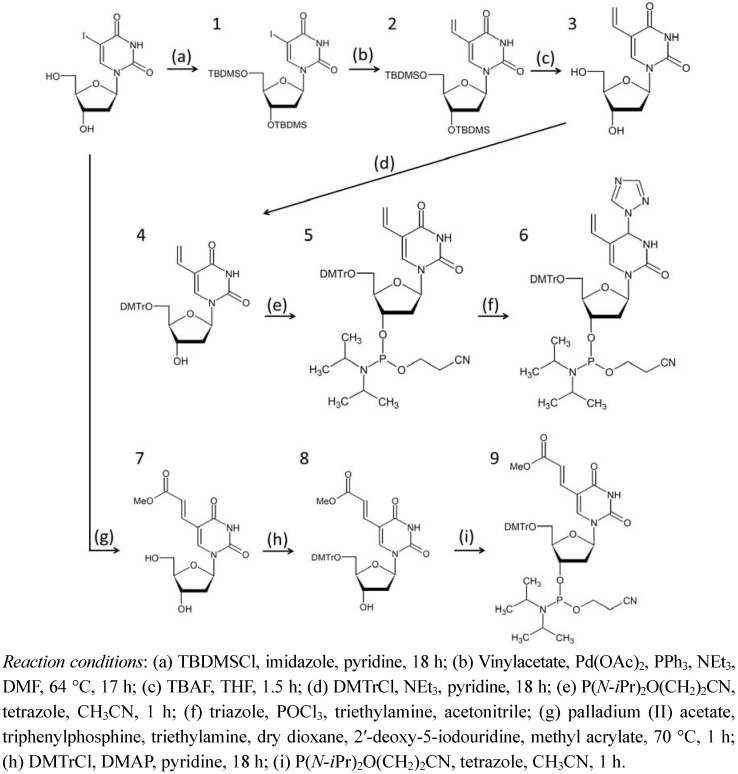
Synthetic scheme of photoresponsive nucleotide.

In a previous study, four of the same ODNs were ligated by photochemical ligation using ^V^U [13]. In this study, we demonstrated the feasibility of ligating four different ODNs using ^V^U and ^V^C (Figure 1A). After we elaborated the ^V^U and ^V^C (Scheme 2), we synthesized ODNs containing ^V^U and ^V^C by automated DNA synthesizer. Figure 1B shows the strategy of reversible photochemical ligation.

**Figure 2:**
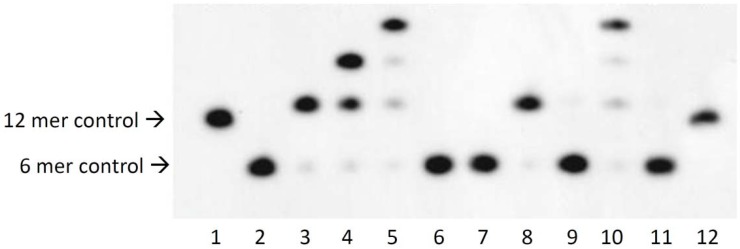
Autoradiogram of a denaturing polyacrylamide gel electrophoresis of photoreaction of ODN 1, ODN 2, ODN 3, ODN 4 and template 1 was annealed and photoirradiated at 366 nm for 8 h at 4 °C. Lane 1, 12-mer control; lane 2, 6-mer control; lane 3, ODN 1 + ODN 2 + template 1 + 366 nm; lane 4, lane 3 + ODN 3; lane 5, Lane 4+ ODN 4; lane 6, lane 5 without 366 nm; lane 7, lane 5 without ODN 2; lane 8, lane 5 without ODN 3; lane 9, lane 5 without template 1; lane 10, lane 5; lane 11, lane 10 + 312 nm.

ODN 1 containing ^32^P at the 5′ end (Experimental section 3.3) and the other ODNs were annealed and irradiated at 366 nm for 8 h and 312 nm for 1 h. Annealing and photoirradiation in detail is shown in experimental section 3.4. The result of denaturing PAGE analysis is shown in Figure 2.

Lane 1 and lane 2 are 12 mer control and 6 mer control respectively. Lane 3, which is ODN 1 and 2, was irradiated at 366 nm for 8 h in the presence of a template. We obtained the 12 mer band ligated ODN 1 and 2. Lane 4 is ODN 3 added to Lane 3. We obtained the 18 mer band ligated ODN 1, 2 and 3. Lane 5 is ODN 4 added to Lane 4. We obtained the 24 mer band ligated ODN 1, 2, 3 and 4. Lane 6 is lane 4 nonirradiated at 366 nm. We obtained the 6 mer band with only ODN 1 ligated. Lane 7, which is ODN 1, 3 and 4, was irradiated at 366 nm in the presence of the template. We obtained the 6 mer band, which is only ODN 1. Lane 8, which is ODN 1, 2 and 4, was irradiated at 366 nm in the presence of the template. We obtained the 12 mer band ligated ODN 1 and 2. Lane 9, which is ODN 1, 2, 3 and 4, was irradiated at 366 nm in the absence of the template. We obtained the 6 mer band, which is only ODN 1. Lane 10 is the same sample as that in lane 5. Lane 11 is lane 10 irradiated at 312 nm for 1 h. We obtained the 6 mer band, which is only ODN 1.

We confirmed that the initial domain becomes longer by comparison with the 2nd domain and 3rd domain. It showed the initial domain made photo cross linking by photoirradiation and this reaction advanced efficiently. This reaction did not proceed without the template and photoirradiation. The photoligated ODNs quantitatively reverted to the original ODNs by irradiation at 312 nm. ^V^U and ^V^C can be ligated in various pairs such as T♢^V^U, T♢^V^C and C♢^V^U by photochemical ligation. So, we extended a ligation pair of only T♢^V^U to four pairs.

**Figure 3:**
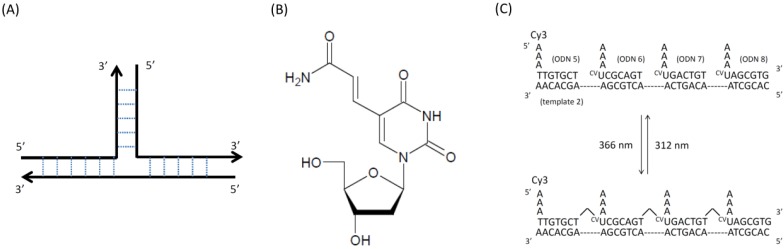
(**A**) Illustrated branched DNA; (**B**) chemical structure of ^CV^U nucleotide; (**C**) schematic illustration for reversible photoligation with^ CV^U and Cy3.

Next, we designed template directed synthetic DNA having branched DNA. Figure 3A shows the branched DNA. Branched DNA has various uses in signal amplification technology and in several types of nanotechnology, such as DNA computing, DNA nanostructures from self-assembled branched units, DNA sensors [17], and nano-electronic devices [18]. We previously reported Multiple-Branched DNA [15] and DNA computing [19] using branched DNA.

**Figure 4:**
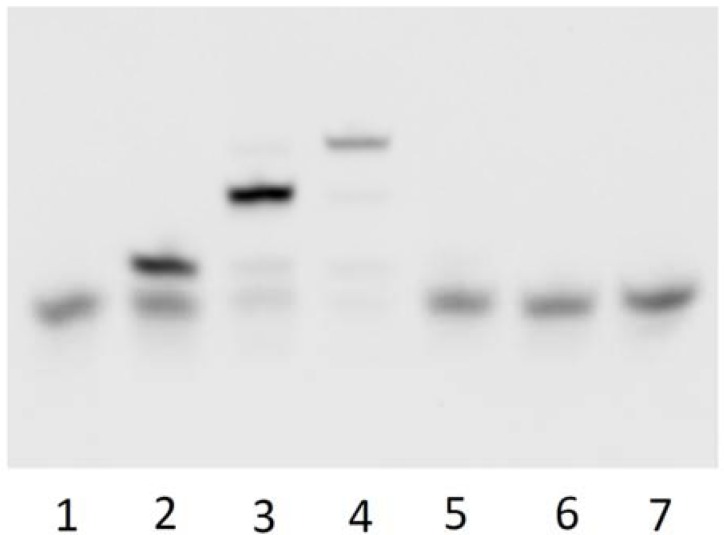
Cy3 fluorescence of a denaturing PAGE of photoreaction of ODN 5, 6, 7 and 8 and template 2 was annealed and photoirradiated at 366 nm for 900 s at 4 °C and 312 nm for 1800 s at room temperature. Lane 1, ODN 5 + template 2 + 366 nm; Lane 2, Lane 1 + ODN 6; Lane 3, Lane 2 + ODN 7; Lane 4, Lane 3 + ODN 8; Lane 5 Lane 4 + 312 nm; Lane 6, Lane 4 without template 2; Lane 7, Lane 4 without 366 nm.

We demonstrated the feasibility of reversible photoligation with ^CV^U. The wavelengths used were 366 nm and 312 nm, the same wavelengths as when using ^V^U. After elaborating the ^CV^U, we synthesized ODNs containing ^CV^U by automated DNA synthesizer. We synthesized ODNs containing ^CV^U inside so we made branched DNA toward developing DNA nanotechnology (Figure 3A). ODN 5 containing Cy3 fluorescence at the 5′ end, ODN 6, ODN 7, ODN 8 and template 2 was annealed and irradiated at 366 nm for 900 s. The result of denaturing PAGE analysis is shown in Figure 4. We obtained Cy3 fluorescence.

Lane 1, which is ODN 5, was irradiated at 366 nm for 900 s in the presence of a template. We obtained the 10 mer band, which is only ODN 5. Lane 2 is ODN 6 added to Lane 1. We obtained the 20 mer band ligated ODN 5 and 6. Lane 3 is ODN 7 added to Lane 2. We obtained the 30 mer band ligated ODN 5, 6 and 7. Lane 4 is ODN 8 added to Lane 3. We obtained the 40 mer band ligated ODN 5, 6, 7 and 8. Lane 5 is lane 4 irradiated at 312 nm for 1800 s. Lane 6, which is ODN 5, 6, 7 and 8, was irradiated at 366 nm for 900 s in the absence template. Lane 7, which is ODN 5, 6, 7 and 8, was nonirradiated at 366 nm. We obtained the 10 mer band, which is only ODN 5 in Lane 5, 6 and 7.

The DNA becomes longer by adding DNA sequentially from the initial domain, which shows that ^CV^U and T are connected by photochemical ligation. This reaction was completely finished for irradiation at 366 nm for 900 s as shown by the results of lane 4. Thus, 900 s is considered sufficient for utilization. And, this reaction did not advance in the absence of a template and nonirradiation at 366 nm. The photoligated ODNs were quantitatively reverted to the original ODNs by irradiation at 312 nm.

**Figure 5:**
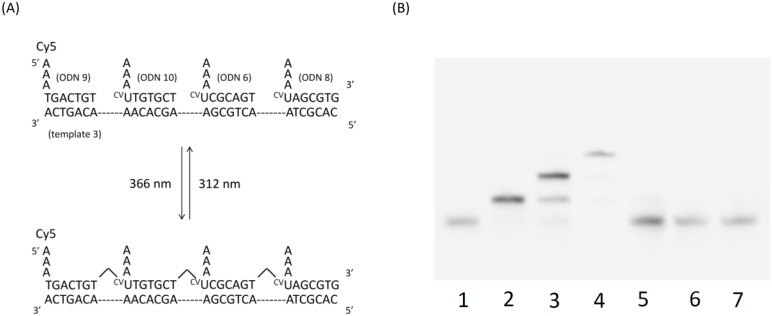
(**A**) Schematic illustration of reversible photoligation with^ CV^U and Cy5; (**B**) Cy5 fluorescence of a Denaturing PAGE of the photoreaction of ODN 9, 10, 6, 8 and template 3 was annealed and photoirradiated at 366 nm for 900 s at 4 °C and 312 nm for 1,800 s at room temperature. Lane 1, ODN 9 + template 3 + 366 nm; Lane 2, Lane 1 + ODN 10; Lane 3, Lane 2 + ODN 6; Lane 4, Lane 3 + ODN 8; Lane 5 Lane 4 + 312 nm; Lane 6, Lane 4 without template 2; Lane 7, Lane 4 without irradiation at 366 nm.

Next, we demonstrated photochemical ligation in other sequences (Figure 5A). ODN 9 is ODN 7 linked Cy5 fluorescence at the 5′ end. The result of denaturing PAGE analysis is shown in Figure 5B. We obtained Cy5 fluorescence.

Lane 1, which is ODN 9, was irradiated at 366 nm for 900 s in the presence of a template. We obtained the 10 mer band, which is only ODN 9. Lane 2 is ODN 10 added to Lane 1. We obtained the 20 mer band ligated with ODN 9 and 10. Lane 3 is ODN 6 added to Lane 2. We obtained the 30 mer band ligated with ODN 9, 10 and 6. Lane 4 is ODN 8 added to Lane 3. We obtained the 40 mer band ligated with ODN 9, 10, 6 and 8. Lane 5 is irradiated at 312 nm to Lane 4 for 1,800 s. Lane 6, which is ODN 9, 10, 6 and 8, was irradiated at 366 nm for 900 s in the absence of a template. Lane 7, which is ODN 9, 10, 6 and 8, was nonirradiated at 366 nm. We obtained the 10 mer band, which is only ODN 9 in Lane 5, 6 and 7. The same as the result above, we confirmed that the ligated product was produced by photochemical ligation, so we can use photochemical ligation regardless of the sequence. As photochemical ligation did not advance without a template, we can use this reaction for sensing the template. Additionally, these results show the difference of reactivity of ^V^U and ^CV^U on photochemical ligation so we can make the system only ^CV^U ligated if photoirradiation was short and ^V^U and ^CV^U was ligated if the photoirradiation time was long by using these compounds respectively.

**Figure 6:**
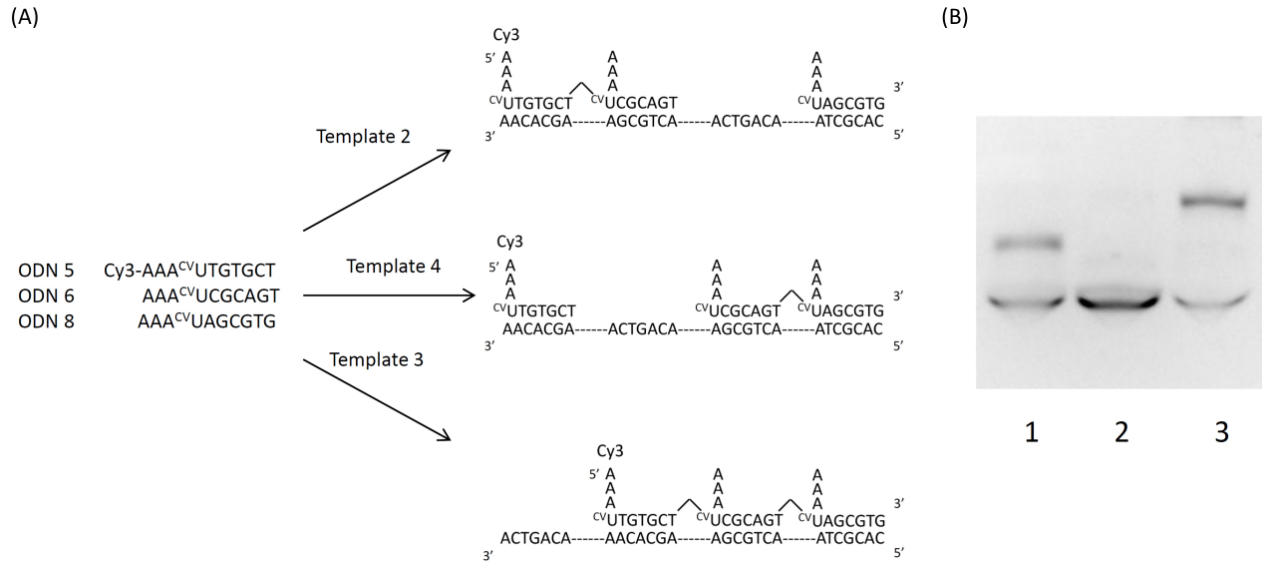
Scheme and denaturing PAGE analysis of a template directed DNA photoligation. (**A**) ODN-5 (10 μM), ODN-6 (10 μM ), ODN-8 (10 μM) and template 2, 4 or 3 (10 μM) irradiation at 366 nm for 1,800 s; (**B**) Cy3 fluorescent of denaturing polyacrylamide gel electrophoresis. Lane 1, template 2; lane 2, template 4; lane 3, template 3.

Next, we demonstrated the template dependence for changing the template in the same three domains (Figure 6A). By using template 2, 3 or 4, we confirmed the size of the ligated product. In the presence of template 2, we should be able to confirm ligated ODN 5 and 6. In the presence of template 4, we should be able to confirm only ODN 5. In the presence of template 3, we should be able to confirm ligated ODN 5, 6 and 8.

ODN 5, 6 and 8 and template 1, 2 or 3 were annealed and photoirradiated at 366 nm. The result of PAGE analysis is shown in Figure 6B. We observed Cy3 fluorescence and it showed the size of the ligation product of ODN 5.

We confirmed that ODN 5 and ODN 6 were ligated in the presence of template 1 and only ODN 5 was ligated in the presence of template 2. In the presence of template 3, ODN 5, 6 and 7 were ligated. This result reflects the template dependence of photochemical ligation. Generally, template-directed DNA ligation can generate a related to the template DNA in a sequence-specific manner. However, this photochemical ligation method is a reversible ligation method, so we can rearrange ODNs using templates. Furthermore, this photochemical ligation is reversible so we can rearrange another sequence after ligated ODN is reverted to original ODNs by irradiation at 312 nm. *In vivo*, DNA works as an information carrier so it is suggested that we can re-edit the information of DNA.

**Figure 7:**
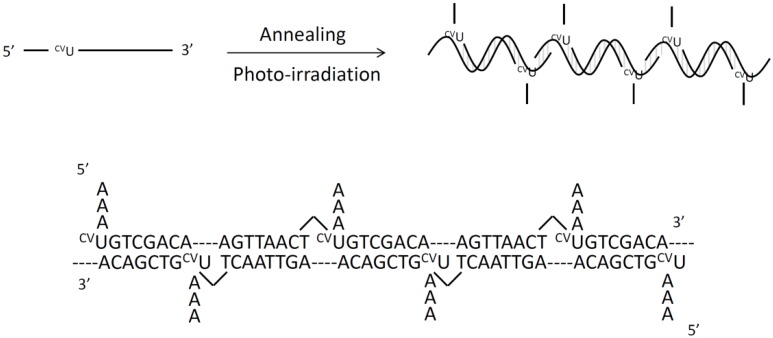
Schematic illustrate of self-assembled structure.

Finally, we researched the activities of photochemical ligation with a self-assembled DNA structure (Figure 7). A self-assembled DNA structure is made by the characteristic of complementarity of DNA such as a holiday junction [20] and DNA origami [21]. Our construction of a self-assembled structure contributes toward developing DNA nanotechnology. We synthesized ODN containing ^CV^U.

**Figure 8:**
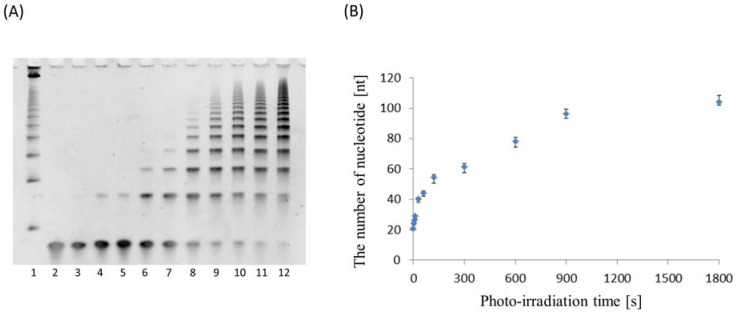
(**A**) Denaturing PAGE analysis of the photoreaction. Lane 1, control 25 bp Ladder Marker; lane 2, non-photoirradiation at 366 nm; lane 3, photoirradiation at 1 s; lane 4, 5 s; lane 5, 10 s; lane 6, 30 s; lane 7, 60 s; lane 8, 120 s; lane 9, 300 s; lane 10, 600 s; lane 11, 900 s; lane 12, 1,800 s; (**B**) The time course of the average of photoligation product at the irradiation time.

ODN 11 was annealed and irradiated at 366 nm from 1 s to 1,800 s at 4 °C. Ligation products should become bigger according to photoirradiation time. The PAGE analysis result is shown in Figure 8A. We confirmed that ligation products become bigger according to photoirradiation time. Samples of 900 s and 1,800 s showed no change so this reaction was completely irradiated at 366 nm for 1,800 s. The size of the final structure became longer by optimization of the sequence. The average of nucleotide photoirradiation time was plotted (Figure 8B). This figure shows that the nucleotide average increases very fast at first and does not change over 900 s. This photochemical ligation is not a bimolecular reaction such as enzyme-substrate [22] but all reactions advance in parallel simultaneously by photoirradiation over the whole reaction field. Moreover, this structure is not a normal double strand but a double strand containing branched DNA. This branched DNA is not made by a ligation enzyme. So, we can modify branched DNA such as biotin and nucleotide aptamer. In a previous study, a molecule was spotted onto DNA origami [23]. We can spot various molecules using this linear structure too.

In this study, it was shown that DNA may be ligated or cut by photo efficiency. This means that it is possible to carry out reorganization collection on the information included in DNA. In our body, the reorganization collection of the DNA is not carried out, but after being transferred as RNA, the reorganization collection is carried out, such as by splicing. Without passing RNA, by carrying out the reorganization collection of the DNA, it might be possible to create the translation process to protein artificially.

## 3. Experimental

### 3.1. General

^1^H-NMR spectra were measured with AVANCE III NMR 400 (Bruker, 400 MHz) spectrometer. Coupling constant (*J* value) are reported in hertz. The chemical shifts are expressed in ppm downfield from tetramethylsilane, using residual chloroform (δ = 7.24 in ^1^H-NMR) and DMSO (δ = 2.49 in ^1^H-NMR) as an internal standard. Mass spectra were recorded on a Voyager-DE PRO-SF, Applied Biosystems. Irradiation was performed by UV-LED (OMRON, ZUV, 366 nm, 1.6 W/cm^2^) or 2 W transilluminator (FUNAKOSHI, TR-312R/J, 312 nm). HPLC was performed on a Chemcobond 5-ODS-H column (10 × 150 mm, 4.6 × 150 mm) or a Chemcosorb 5-ODS-H column (4.6 × 150 mm) with a JASCO PU-980, HG-980-31, DG-980-50 system equipped with a JASCO UV 970 detector at 260 nm. The reagents for the DNA synthesizer such as A, G, C, T-β-cyanoethyl phosphoramidite, and CPG support were purchased from Glen Research. Calf intestine alkaline phosphatase (AP) was purchased from Promega. Nuclease P1 was purchased from Yamasa.

### 3.2. Synthesis of Artificial Nucleotide

*3′,5′-Bis-*O*-*tert*-butyldimethysilyl-5-iodo-2′-deoxyuridine* (**1**). 5-Iodo-2′-deoxyuridine (2.504 g, 7.071 mmol) was dissolved in dry pyridine (10 mL) and concentrated to dryness in *vacuo* three times, Imidazole (1.462 g, 21.47 mmol) and *t*-butyldimethylsilyl chloride (3.222 g, 21. 38 mmol) were added to a solution of 5-iodo-2′-deoxyuridine (2.504 g, 7.071 mmol) in dry pyridine (35 mL). The solution was stirred at ambient temperature under nitrogen atmosphere for 18 h. The reaction was monitored by TLC (Hexane/EtOAc, 4:1), which showed the absence of starting material. After evaporation *in vacuo*, the residue was extracted with ethyl acetate (EtOAc) (100 mL × 3) and water (100 mL). The organic layer was collected, dried over anhydrous magnesium sulfate, filtered, and evapolated to dryness *in vacuo*. The crude product was purified by silica gel column from the column with hexane/EtOAc (4:1) as eluted to give 1 (3.587 g, 87%) as a white solid. ^1^H-NMR (400 MHz CDCl_3_) δ 0.056 (s, 3H, CH_3_Si), 0.065 (s, 3H, CH_3_Si), 0.13 (s, 3H, CH_3_Si), 0.14 (s, 3H, CH_3_Si), 0.88 (s, 9H, *t*-BuSi), 0.93 (s, 9H, *t*-BuSi), 1.97 (ddd, *J* = 13.2 Hz, *J* = 7.7 Hz, *J* = 2.0 Hz, 1H, H-2′β), 2.28 (ddd, *J* = 13.2 Hz, *J* = 7.7 Hz, *J* = 2.0 Hz, 1H, H-2′α ), 3.73 (dd, *J* = 11.4 Hz, *J* = 2.4 Hz. 1H, H-5′), 3.87 (dd, *J* = 11.4 Hz, *J* = 2.4 Hz, 1H, H-5′), 3.98–3.96 (m, 1H, H-4′), 4.39–4.36 (m, 1H, H-3′′), 6.25 (dd, *J* = 8.2 Hz, *J* = 5.7 Hz, 1H, H-1′), 8.05 (brs., 1H, NH), 8.07 (S, 1H, H-6). FAB MS *m/e* 583[(M+H)^+^], HRMS: (positive ion FAB) calcd for C_21_H_39_IN_2_O_2_Si_2_ [(M+H)^+^] 5.82.1444, found 583.1534.

*3′,5′-Bis-*O*-*tert*-butyldimethylsily-5-vinyl-2′-deoxyuridine* (**2**). Palladium (II) acetate (0.125 g, 0.557 mmol), triphenylphosphine (0.250 g, 0.953 mmol), and anhydrous triethylamine (6.60 mL, 47. 35 mmol) were combined in anhydrous dimethylformamide (19 mL) and stirred at 64 °C until an intense red color developed. 3′,5′-Bis-*O*-*tert*-butyldimethysilyl-5-vinyl-2′-deoxyuridine (3.566 g, 6.12 mmol) and vinyl acetate (30.0 mL, 325.5 mmol) dissolved in anhydrous dimethylformamide (30 mL) were then added, and stirred at 64 °C until nitrogen atmosphere for 17 h. TLC analysis of the reaction mixture in hexane/EtOAc (4:1) showed the absence of starting material (Rf = 0.24) and the formation of the product (Rf = 0.29). The reaction mixture was filtered to remove the resulting precipitate, and the filtered was evaporated to dryness in *vacuo*, then extracted with ethyl acetate (40 mL × 3) and water (40 mL). The organic layer was collected, dried over anhydrous magnesium sulfate, filtered, and evaporated to dryness under reduced pressure, the crude product was product was purified by silica gel column chromatography. 3′,5′-Bis-*O*-*tert*-butyldimethysilyl-5-vinyl-2′-deoxyuridine eluted from the column with hexane/EtOAc (4:1) was isolated in 55.9% yield (1.651 g) as a yellow viscous oil. ^1^H-NMR (400 MHz, CDCl_3_) δ 0.056 (s, 6H, CH_3_Si), 0.065 (s, 6H, CH_3_Si), 0.079 (s, 6H, CH_3_Si ×2), 0.88 (s, 9H, *t*-Bu), 0.89 (s, 9H, *t*-Bu), 1.99 (ddd, *J* = 13.2 Hz, *J* = 7.7 Hz, *J* = 5.8 Hz, 1H, H-5′), 2.29 (ddd, *J* = 13.2 Hz, *J* = 5.6 Hz, *J* = 2.5 Hz, 1H, H-5′), 3.96–3.94 (m, 1H, H-4′), 4.37–4.40 (m, 1H, H-3′), 5.23 (dd, *J* = 11.3 Hz, *J* = 1.5 Hz, 1H, vinyl trans), 5.98 (dd, *J* = 17.4 Hz, *J* = 1.5 Hz, 1H, vinyl cis), 6.25–6.29 (m, 1H, H-1′), 6.33 (dd, *J* = 17.4 Hz, *J* = 11.3 Hz, 1H, CH=CH_2_), 7.65 (s, 1H, H-6), 8.02 (s, 1H, NH). FAB MS: *m/e* 483 [(M+H)^+^], HRMS: (positive ion FAB) calcd for C_23_H_42_N_2_O_5_Si_2 _[(M+H)^+^] 482.2632, found 483.2686.

*5-Vinyl-2′-deoxyuridine* (**3**). To a THF solution of 3′,5′-bis-*O*-*tert*-butyldimethylsilyl-5-vinyl-2′-deoxyuridine (1.613 g, 3.34 mmol) was added tetrabuylammonium fluoride (10.0 mL of 1 M THF solution, 10.0 mmol) and the solution was stirred at ambient temperature for 1.5 h. The solvent was removed under reduced pressure and the residue was purified by silica gel column chromatography. 5-Vinyl-2′-deoxyuridine eluted from the column with CHCl_3_/MeOH (8:1) was isolated in 92.3% yield (0.784 g) as a yellow oil. ^1^H-NMR(400 MHz, DMSO-*d*_6_) δ 2.11–2.15 (m, 2H, H-2′), 3.43–3.65 (m, 2H, H-5′), 3.77–3.79 (m, 1H, H-4′), 4.24–4.26 (m, 1H, H-3′), 5.09–5.26 (m, 3H, vinyl *trans*, 3′-OH and 5′-OH), 5.91 (dd, *J* = 17.3 Hz, *J* = 2.0 Hz, 1H, vinyl cis), 6.15 (t, *J* = 6.6 Hz, 1H, H-1′), 6.36 (dd, *J* = 17.3 Hz, *J* = 11.5 Hz, 1H, CH=CH_2_),8.11 (s, 1H, H-6), 11.41 (bs, 1H, NH).

*5′-*O*-(4,4′-Dimethoxytriryl)-5-vinyl-2′-deoxyuridine* (**4**). 5-Vinyl-2′-deoxyuridine (0.630 g, 2.48 mmol) was dissolved in dry pridine and coevaporated three times. 4,4′-Dimethoxytrityl chloride (0.913 g, 2.69 mmol), *N,N*-dimethylaminopyridine (19 mg, 0.156 mmol) and triethylamine (0.375 mL, 2.69 mmol) was added to a solution of 5-vinyl-2′-deoxyuridine in dry pyridine (16 mL) The solution was stirred at ambient temperature under nitrogen atmosphere 18 h. The TLC analysis CHCl_3_/MeOH (8:1) showed the presence of starting material, but the reaction mixture was evaporated to dryness *in vacuo*. The residue was extracted with CHCl_3_ (20 mL × 3) and water (20 mL), and the organic layer was collected, dried over Na_2_SO_4_, filtered, and evapolated to dryness under reduced pressure. The crude product was purified by silica gel column chromatography with CHCl_3_/MeOH (8:1) and 5′-*O*-(4,4′-dimethoxytrityl)-5-vinyl-2′-deoxyuridine (1.26 g) was isolated in 75.0% yield as a white solid. ^1^H-NMR (400 MHz, CDCl_3_) δ 2.29 (ddd, *J* = 13.6 Hz, *J* = 7.2 Hz, *J* = 6.1 Hz, 1H, H-2β), 2.41 (ddd, *J* = 13.6 Hz, *J* = 6.1 Hz, *J* = 3.4 Hz, 1H, H-2′α), 3.37 (dd, *J* = 10.4 Hz, *J* = 3.5 Hz, 1H, H-5′), 3.45 (dd, *J* = 10.4 Hz, *J* = 3.5 Hz, 1H, H-5′), 3.77 (s, 6H, OCH_3_ ×2), 4.02–4.05 (m, 1H, H-4′), 4.52–4.56 (m, 3H, H-3′), 4.93 (dd, *J* = 10.3 Hz, *J* = 2.6 Hz, 1H, vinyl *trans*), 5.71 (dd, *J* = 10.3 Hz, *J* = 17.8 Hz, 1H, vinyl *cis*), 5.78 (dd, *J* = 2.6 Hz, 17.6 Hz, 1H, vinyl *cis*), 6.35 (dd, *J* = 7.2 Hz, *J* = 6.1 Hz, 1H, H-1′), 6.80–6.98 (m, 4H, H-*ortho* to OCH_3_ ×4), 7.22–7.30 (m, 9H, phenyl), 7.64 (s, 1H, H-6), 7.91 (bs, 1H, NH). FAB MS: *m/e* 557 [(M+H)^+^], HRMS: (positive ion FAB) calced for C_32_H_33_N_2_O_7_ [(M+H)^+^] 557. 62, found 557.228.

*5′-*O*-(4,4′-Dimethoxytrityl)-3′-*O*-[2-cyanoethoxy-(N,N-diisopropylamino)-phosphino]-5-vinyl-2′-deoxyuridine* (**5**). 5′-*O*-(4,4′-Dimethoxytriryl)-5-vinyl-2′-deoxyuridine (166 mg, 0.298 mmol) in a sealed bottle with septum was dissolved in dry acetonitrile and coevaporated three times in *vacuo*. After substitution with argon, 2-cyanoethyl-*N,N,N′,N′-*tetraisopropylphosphoroamidite (99.0 μL, 0.309 mmol) in dry acetonitrile (2.0 mL), and 0.5 M tetrazole in dry acetonitrile were stirred for 1.0 h. After the completion of the reaction as evidence by TLC, the reaction mixture was extracted with ethyl acetate (20 mL × 2), which was washed with saturated sodium bicarbonate aqueous solution and water (15 mL). The organic layer was collected, dried over anhydrous sodium sulfate, filtered, and evaporated to dryness under reduced pressure. Then, the crude product 5′-*O*-(4,4′-Dimethoxytrityl)-3′-*O*-[2-cyanoethoxy-(*N,N*-diisopropylamino)-phosphino]-5-vinyl-2′-deoxyuridine (228 mg) in a sealed bottle with septum was dissolved in dry acetonitrile and coevaporated three times and was used in automated DNA synthesizer without further purification.

*5′-*O*-(4,4′-Dimethoxytrityl)-N^4^-1,2,4-triazolyl-3′-*O*-[2-cyanoethoxy-(N,N-diisopropylamino)-phosphino]-5-vinyl-2′-deoxyuridine* (**6**). To an ice cooled stirred suspension of 1,2,4-triazole (1.52 g, 22.0 mmol) in dry acetonitrile (28 mL) was added slowly POCl_3_ (0.44 mL, 4.72 mmol) followed by dry triethylamine (3.33 mL, 23.9 mmol). After 30 min a solution of 5′-*O*-(4,4′-dimethoxytrityl)-3′-*O*-(2-cyanoethoxy-(*N,N*-diisopropylamino)-phosphino)-5-vinyl-2′-deoxyuridine (290 mg, 0.359 mmol), in dry acetonitrile (3 mL) was added over a period of 5 min. Ice-cooled stirring was continued for 20 min followed by 40 min at room temperature. The reaction solution was diluted with EtOAc (50 mL) and extracted with saturated NaHCO_3_ solution (50 mL). The organic layer was collected, dried over anhydrous sodium sulfate, filtered, and evaporated to dryness to yield 5′-*O*-(4,4′-dimethoxytrityl)-*N^4^*-1,2,4-triazolyl-3′-*O*-[2-cyanoethoxy-(*N,N*-diisopropylamino)-phosphino]-5-vinyl-2′-deoxyuridine (276 mg, 0.341 mmol, 95%), which was directly used in an automated DNA synthesizer without further purification. ^31^P-NMR(CDCl_3_, 85% H_3_PO_4_ in D_2_O ext. o ppm) δ 149.811, 150.386 (diastereomer of the product).

*5-(2-Carboxymethoxyvinyl)-2′-deoxyuridine* (**7**). A mixture of palladium (II) acetate (0.15 g, 0.90 mmol), triphenylphosphine (0.37 g, 1.90 mmol) and triethylamine (2.5 mL, 18 mmol) in dry dioxane (25 mL) was stirred at 70 °C until an intense red color had developed. To this there was then added 2′-deoxy-5-iodouridine (5.0 g, 14 mmol) and methyl acrylate (2.35 g, 27.0 mmol) and the mixture was refluxed for 1 h. It was then filtered while still hot and evaporated *in vacuo*. The crude product was purified by column chromatography (4% MeOH/EtOAc) to give 5-(2-carboxymethoxyvinyl)-2′-deoxyuridine (3.1 g, 70%) as a white powder. ^1^H-NMR (400 MHz, DMSO-*d*6) δ 2.14–2.18 (m, 2H, H-2′), 3.54–3.64 (m, 2H, H-5′), 3.67 (s, 3H, OCH_3_), 3.77–3.80 (m, 1H, 4′-OH), 4.23–4.27 (m, 1H, H-3′), 5.16 (bs, 1H, 3′-OH), 5.25 (bs, 1H, 5′-OH), 6.84 (d, *J* = 16 Hz, 1H, alpha vinyl), 7.36 (d, *J* = 16 Hz, 1H, beta vinyl), 8.41 (s, 1H, H-6), 11.63 (bs, 1H, NH). HRMS (positive ion FAB) calcd for C_13_H_17_O_7_N_2_ [(M+H)^+^] 313.1036, found 313.1034.

*5′-*O*-(4,4′-Dimethoxytriryl)-5-(2-Carboxymethoxyvinyl)-2′-deoxyuridine* (**8**). 5-(2-Carboxymethoxyvinyl)-2′-deoxyuridine (1.210 g, 3.87 mmol) was dissolved in dry pyridine and coevaporated three times. 4,4′-Dimethoxytrityl chloride (1.575 g, 4.64 mmol), *N,N*-dimethylaminopyridine (30.2 mg, 0.248 mmol) was added to a solution of 5-vinyl-2′-deoxyuridine in dry pyridine (30 mL). The solution was stirred at ambient temperature under nitrogen atmosphere 18 h. The TLC analysis (CHCl_3_/MeOH 9:1) showed the presence of starting material, but the reaction mixture was evaporated to dryness in vacuo. The crude product was purified by silica gel column chromatography with CHCl_3_/MeOH (8:1), and 5′-*O*-(4,4′-dimethoxytrityl)-5-vinyl-2′-deoxyuridine (1.67 g) was isolated in 70.0% yield as a yellow solid. ^1^H-NMR (400 MHz, CDCl_3_) δ 2.25–2.35 (m, 2H, H-2′α, H-2′β), 2.52–2.57 (m, 1H, H-3′), 3.38–3.48 (m, 2H, H-1′, H-4′), 3.65 (s, 3H, OCH_3_), 3.77 (d, 6H, *J* = 1.2 Hz, OCH_3_ ×2), 4.52 (s, 1H, 3′-OH), 6.28 (dd, *J* = 6.4 Hz, 2.4 Hz, 1H, H-1′), 6.95–6.68 (m, 4H, H-*ortho* to OCH_3_ ×4), 6.92–6.96 (m, 2H, alpha and beta vinyl), 7.20–7.30 (m, 9H, phenyl), 7.86 (s, 1H, NH), 8.86 (s, 1H, H-6). HRMS (positive ion FAB) calcd for C_34_H_34_N_2_O_9_ [(M+H)^+^] 615.2342, found 615.2345.

*5′-*O*-(4,4′-Dimethoxytriryl)-3′-*O*-[2-cyanoethoxy-(*N,N*-diisopropylamino)-phosphino]-5-(2-carboxy-methoxyvinyl)-2′-deoxyuridine* (**9**). 5′-*O*-(4,4′-Dimethoxytriryl)-5-(2-carboxymethoxyvinyl)-2′-deoxyuridine (183 mg, 0.298 mmol) in a sealed bottle with septum was dissolved in dry acetonitrile and coevaporated three times *in vacuo*. After substitution with argon, 2-cyanoethyl-*N,N,N′,N′-*tetraisopropyl-phosphoroamidite (99.0 μL, 0.309 mmol) in dry acetonitrile (2.0 mL), 0.5 M tetrazole in dry acetonitrile were stirred for 1.0 h. After the completion of the reaction as evidence by TLC, the reaction mixture was extracted with ethyl acetate (20 mL × 2), which was washed with saturated sodium bicarbonate aqueous solution and water (15 mL). The organic layer was collected, dried over anhydrous sodium sulfate, filtered, and evaporated to dryness under reduced pressure. Then, the crude product 5′-*O*-(4,4′-dimethoxytrityl)-3′-*O*-[2-cyanoethoxy-(*N,N*-diisopropylamino)-phosphino]-5-vinyl-2′-deoxyuridine (228 mg) in a sealed bottle with sepum was dissolved in dry acetonitrile and coevaporated three times and was used for automated DNA synthesizer without further purification.

### 3.2. Synthesis of Oligonucleotides in This Experiment

Oligonucleotides were prepared by β-(cyanoethyl) phosphoramidite method on controlled pore glass supports (1 mmol) by using ABI3400 DNA synthesizer. Cyanoethylphosphoramidite of elaborated compounds was prepared as described above. After automated synthesis, the oligomer was detached from the support by soaking in conc. aqueous ammonia for 1 h at room temperature. Deprotection was conducted by heating the conc. aqueous for 10 h at 55 °C conc. aqueous ammonia was then removed by speedvac, and the crude oligomer was purified by reverse phase HPLC and lyophilized. The Purity and concentration of all oligonucleotides were determined by complete digestion with s.v. PDE, P-1 nuclease, and AP to 2′-deoxymononucleosides. DNA base sequence was shown in Table 1.

**Table 1:** Sequence of using oligonucleotide.

No.	Sequence(5′-3′)
ODN 1	TGTGCT
ODN 2	^V^CGCAGT
ODN 3	^V^UTTCCC
ODN 4	^V^UGCGTG
ODN 5	Cy3-AAATTGTGCT
ODN 6	AAA^CV^UCGCAGT
ODN 7	AAA^CV^UGACTGT
ODN 8	AAA^CV^UAGCGTG
ODN 9	Cy5-AAATGACTGT
ODN 10	AAA^CV^UTGTGCT
ODN 11	AAA^CV^UGTCGACAAGTTAACT
Template 1	CACGCAGGGAAAACTGCGAGCACA
Template 2	CACGCTAACAGTCAACTGCGAAGCACAA
Template 3	CACGCTAACTGCGAAGCACAAACAGTCA
Template 4	CACGCTAACTGCGAACAGTCAAGCACAA

### 3.3. Preparation of 5′-^32^P-end-labeled ODN

The Oligodeoxynucleotides (ODNs 400 pmol) were 5′-end-labeled by phosphorylation with 4 μL of [γ-^32^P] ATP and 4 μL of T4 polynucleotide kinase using standard procedures [24]. The 5′-end-labeled ODNs were recovered by ethanol precipitation and further purified by 15% denaturing gel electrophoresis and isolated by the crush and soak method [25].

### 3.4. Annealing and Photoirradiation

In the ^V^U and ^V^C, a reaction mixture (total volume 10 μL) containing initial ODN (10μM), 2nd ODN (11 μM), 3rd ODN (12 μM), 4th ODN (13 μM) and template (10 μM) in 50 mM Sodium cacodylate buffer (pH 7.0) in an Eppendorf tube was irradiated at 366 nm at 4 °C with transilluminator and 312 nm at room temperature with transilluminator (Funakoshi, 2 mW/cm^2^). In the ^CV^U, a reaction mixture (total volume 20 μL) containing initial ODN (10 μM), 2nd ODN (11 μM), 3rd ODN (12 μM), 4th ODN (13 μM) and template (10 μM) in 50 mM Sodium cacodylate buffer (pH 7.0) in an Eppendorf tube was cooled from 90 °C to 4 °C over 72 h by thermal cycler. Next, an annealed mixture was irradiated at 366 nm at 4 °C with LED (Omron, 1.6 W/cm^2^) and 312 nm at room temperature with transilluminator (Funakoshi, 2 mW/cm^2^).

### 3.5. Photoirradiation of DNA Oligomer as Monitored by PAGE

In the ^V^U and ^V^C, to the reaction mixture (total volume 10 μL) 10 μM of loading buffer (a solution of 80% v/v formamide 1mM EDTA, 0.1% xylene cyanol, and 0.1% bromophenol blue) was added to quench the reaction and the samples (1–2 μL, ca 2–4 × 10^3^ cpm) were loaded onto 15% (19:1) polyacrylamide 7M urea denaturing gel and electrophoresed at 700 V for 30 min. The gel was dried and exposed to X-ray film with an intensifying sheet at −80 °C. In the ^CV^U, the reaction mixture was diluted with 8 M Urea in formamide 10 times. To this diluted solution (3 μL) containing initial ODN (200 nM), 2nd ODN (220 nM), 3rd ODN (240 nM), 4th ODN (260 nM) and template (200 nM) 3 μL of loading buffer (36% glycerol and 30 mM EDTA) were added. The solution was loaded onto 15% (19:1) polyacrylamide, 8M Urea and 25% formamide denatured gel and electrophoresed at 150 V for 120 min. The gel was exposed to Cy3 or Cy5 fluorescence.

### 3.6. Time Course of the Self-Assembled Structure by PAGE

A reaction mixture (total volume 20 μL) containing ODN 11 (25 μM) and 5 mM Magnesium chloride in 50 mM sodium cacodylate buffer (pH 7.0) in an Eppendorf tube was cooled 90 °C for 5 min and from 70 °C to 4 °C over 72 h by thermal cycler. This annealed mixture was irradiated at 366 nm at 4 °C with LED (Omron, 1.6 W/cm^2^) for 0 s, 1 s, 5 s, 10 s, 30 s, 60 s, 120 s, 300 s, 600 s, 900 s and 1800 s. This reaction mixture was diluted with 8 M urea in formamide 10 times. To this diluted solution (3 μL) containing ODN 11 (500 nM) 3 μL of loading buffer (36% glycerol, 30 mM EDTA, 0.05% xylene cyanol and 0.05% bromophenol blue) was added. The solution was loaded onto 15% (19:1) polyacrylamide, 8 M Urea and 25% formamide denatured gel and electrophoresed at 150 V for 120 min. The gel was observed by SYBR Gold.

## 4. Conclusions

We demonstrated that ^V^U, ^V^C and ^CV^U can be used in photochemical ligation. In the study using ^V^U and ^V^C, we succeed in ligating three pairs: T♢^V^U, T♢^V^C and C♢^V^U by [2+2] photocyclization. We can extend the thymine dimer to T♢^V^U, T♢^V^C and C♢^V^U. The reactivity of ^CV^U is very high so the reaction of ^CV^U advances quickly with high efficiency. This photochemical ligation did not advance without a template and a change of alignment sequence by the sequence of the template and therefore we can confirm the existence and sequence of the template. Finally, we made a self-assembled structure for researching photochemical ligation. We confirmed that all reactions advanced simultaneously by photoirradiation in parallel across the whole of the reaction field. In addtion, it is possible to spot various molecules periodically by using this structure containing branched DNA.
